# Graph Neural Networks vs. Traditional QSAR: A Comprehensive Comparison for Multi-Label Molecular Odor Prediction

**DOI:** 10.3390/molecules30234605

**Published:** 2025-11-30

**Authors:** Tengteng Wen, Xianfa Cai, Jincheng Li

**Affiliations:** Guangdong College of Medical Information Engineering, Pharmaceutical University, Guangzhou 510006, China; wentengteng@gdpu.edu.cn (T.W.); xianfacai@gdpu.edu.cn (X.C.)

**Keywords:** molecular odor prediction, graph neural network (GNN), multi-label classification, threshold optimization

## Abstract

Molecular odor prediction represents a fundamental challenge in computational chemistry with significant applications in fragrance design, food science, and chemical safety assessment. While traditional Quantitative Structure–Activity Relationship (QSAR) methods rely on hand-crafted molecular descriptors, recent advances in graph neural networks (GNNs) enable direct end-to-end learning from molecular graph structures. However, systematic comparison between these approaches for multi-label odor prediction remains limited. This study presents a comprehensive evaluation of traditional QSAR methods compared with modern GNN approaches for multi-label molecular odor prediction. Using the GoodScent dataset containing 3304 molecules with six high-frequency odor types (fruity, green, sweet, floral, woody, herbal), we systematically evaluate 23 model configurations across traditional machine learning algorithms (Random Forest, SVM, GBDT, MLP, XGBoost, LightGBM) with three feature-processing strategies and three GNN architectures (GCN, GAT, NNConv). The results demonstrate that GNN models achieve significantly superior performance, with GCN achieving the highest macro F1-score of 0.5193 compared to 0.4766 for the best traditional method (MLP with basic preprocessing), representing a 24.1% relative improvement. Critically, we discover that threshold optimization is essential for multi-label chemical classification. These findings establish GNNs as the preferred approach for molecular property prediction tasks and provide crucial insights for handling class imbalance in chemical informatics applications.

## 1. Introduction

Molecular odor prediction stands as one of the most challenging problems in machine olfaction, bridging the gap between molecular structure and human sensory perception. The ability to predict olfactory properties from a chemical structure has profound implications for fragrance and flavor industries, where the development of new compounds traditionally relies on expensive and time-consuming experimental screening processes [1].

The lack of high-quality, large-scale data is a critical issue in molecular odor prediction. To build a high-quality database for molecular odor perception, there are several obstacles. First, the perception of an odor is subjective by sniffers since individuals have different olfactory sensitivities and knowledge background, although sniffers can be well-trained to identify and evaluate the odor types subjectively [2,3]. Second, the labeling of odor types is difficult to standardize since different regions have different notions and cultures, which makes it difficult to build a comprehensive and standardized database [4,5]. Third, the diversity of corpus for flavor and fragrance profiles leads to the sparsity of odor labels. In recent years, the effort to accumulate and unify the olfactory perception corpus has been ongoing. Olfaction-based databases such as OlfactionBase and Pyrfume have been established which promote the development of molecular odor prediction [6,7,8].

**Figure 1:**
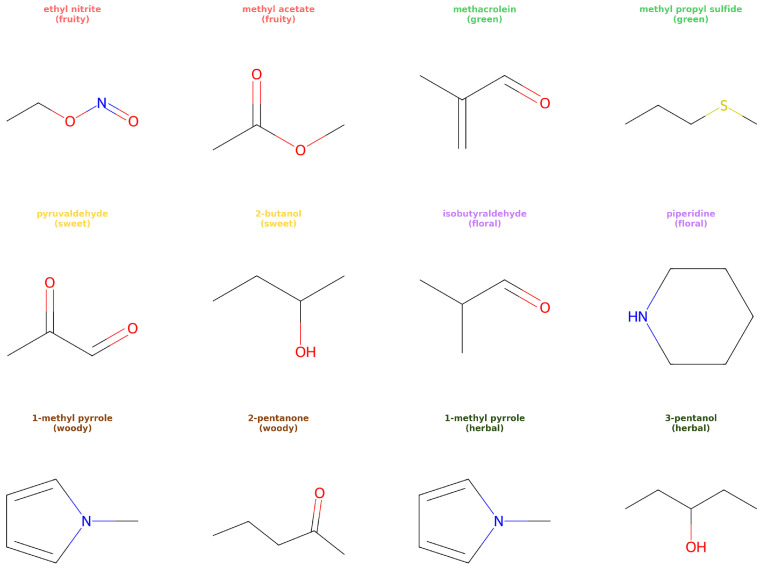
Representative chemical structures from the GoodScent dataset with six odor categories (fruity, green, sweet, floral, woody, herbal).

The relationship between molecular structure and odor perception involves complex interactions between chemical compounds and olfactory receptors, making it inherently difficult to model computationally. Figure 1 illustrates representative examples from GoodScent, showing different structural features with distinct odor characteristics. Traditional approaches have relied on Quantitative Structure–Activity Relationship (QSAR) methods, which use hand-crafted molecular descriptors to capture structural features relevant to biological activity. However, these methods suffer from several limitations: they require domain expertise for feature engineering, may miss important structural patterns, and often struggle with the multi-label nature of odor classification. Traditional linear methods such as principal component analysis (PCA) or linear discriminant analysis (LDA) rely heavily on selected molecular features since these linear methods may suffer from the curse of dimensionality and their inability to capture the complex non-linear relationships inherent in molecular data. More and more non-linear methods such as Random Forest (RF), gradient boosting decision trees (GBDTs), and extreme gradient boosting (XGBoost) have been used for molecular property prediction tasks in this area [9,10]. Automatic feature selection methods employed in these non-linear methods can help to select the most relevant features for the prediction task [11].

Recent advances in deep learning, such as convolutional neural networks (CNNs) and graph neural networks (GNNs), offer a promising alternative by enabling direct learning from molecular graph structures [12,13,14,15]. SchNet is a deep neural network based on continuous-filter convolutions that learns rotationally, translationally, and permutationally invariant representations directly from atomic types and coordinates, achieves ab initio accuracy in molecular and materials property prediction, potential energy surface fitting, and the construction of efficient force fields, and has for the first time enabled nanosecond-scale path-integral molecular dynamics simulations of the $C_{20}$ fullerene [16]. GNNs can automatically discover relevant molecular patterns without requiring explicit feature engineering, potentially capturing complex structure–activity relationships that traditional descriptors might miss. Despite the theoretical advantages of GNNs, there has been limited systematic comparison with established QSAR methods for odor prediction. Most existing studies focus on single-label classification or evaluate only a subset of available methods, making it difficult to draw definitive conclusions about the relative merits of different approaches [12,17]. DimeNet explicitly incorporates the directions between atom pairs as rotation-equivariant messages and uses spherical Bessel functions combined with spherical harmonics to jointly encode interatomic distances and angles, achieving a further reduction in errors on QM9 and MD17 by 31% and 76%, respectively, while satisfying molecular dynamics requirements of twice-differentiable energies and conservative forces [18]. J. Cremer et al. proposed an SE(3)-equivariant graph Transformer (ET) that directly applies to 3D conformations, achieving AUC scores on 11 toxicity datasets that are comparable to or better than the 2D state of the art [19]. Other research focuses on using a combination of GNNs and other methods to improve the prediction performance [20].

Deep learning methods such as GNNs have realized end-to-end structure-to-property/kinetics/synthesis learning. The next step will require an integrated breakthrough that unifies big data, symmetry constraints, and interpretability [21]. Amir K. et al. introduced interpretable graph neural networks (GNNs) for the first time into the prediction of carbon K-edge X-ray absorption spectra (XAS), using time-dependent density functional theory (TDDFT)-computed “atomic–orbital contributions” as an interpretability benchmark [22]. This work demonstrates that only architectures that simultaneously capture local and global information, such as GATv2 and GraphNet, can both accurately predict spectral profiles and yield peak assignments consistent with quantum–chemical intuition. Dmytro S. proposed a learnable adaptive thresholding layer based on “global–local signal fusion,” which measures the global rarity of each label using inverse document frequency (IDF) and the local neighborhood consistency of each sample using KNNs and fuses the two into a per-label, per-sample threshold penalty term added to the loss [23]. A lightweight MLP augmented with this mechanism achieves a macro F1 of 0.1712 on AmazonCat-13K, substantially outperforming existing tree-based and Transformer-based methods, while remaining plug-and-play and easily interpretable. Chirag A. et al. introduced a programmable synthetic graph generator, ShapeGGen, together with a companion evaluation library, GraphXAI, to provide the study of GNN explainability with benchmark datasets endowed with ground-truth explanations and free of known pitfalls, along with a unified evaluation framework [24].

The other critical issue in molecular odor prediction is the imbalance of odor type labels which may lead to the overfitting of prediction models. One of the reasons is that there is no common standard for labeling the odor types. Most of the labels for odors come from the definitions in human language, and there is no universal standard, so the resulting arbitrariness leads to a proliferation of labels. The sparsity of odor labels leads to the unbalanced distribution of odor type labels. From the perspective of informatics, odor labels have the characteristics of multi-label classification, based on the pattern theory of the olfactory system and the emotional and cognitive processing of odor labels in psychology [25]. Accordingly, building the odor prediction models based on multi-label classification methods is a promising approach. The work of this paper mainly explores the construction of an odor prediction model based on graph neural networks to improve the prediction ability of imbalanced labels. We also provide a comparison of traditional QSAR methods with modern graph neural network approaches for multi-label molecular odor prediction. Our contributions include the following:

- Systematic evaluation of 23 model configurations across traditional ML and GNN approaches.
- Threshold optimization is a critical technique for multi-label odor type prediction.
- Graph neural networks outperform traditional QSAR methods for multi-label odor type prediction.

The remainder of this paper is organized as follows: Section 4 presents our methodology including problem formulation, traditional QSAR approaches, graph neural network architectures, and threshold optimization strategies. Section 4 describes the experimental setup including dataset preprocessing and training strategies. Section 4 presents the results covering overall model performance comparison, per-label analysis, and threshold optimization impact. Section 3 discusses the implications of our findings and concludes with future research directions.

## 2. Results

### 2.1. Overall Model Performance Comparison

We systematically evaluated all models using identical train/validation/test splits to ensure a fair comparison. The comprehensive results across 23 model configurations reveal significant performance differences between traditional QSAR approaches and graph neural network methods, with GNN models demonstrating superior performance across all evaluation metrics.

Table 1 shows that the achieved macro F1-score of 0.5193 represents a significant advancement in computational olfaction. This performance level is particularly noteworthy considering the inherent challenges of multi-label odor prediction: (1) the complex many-to-many mapping between molecular structure and olfactory perception, (2) severe class imbalance across odor types (ranging from 12.89% to 30.93%), and (3) the limited size of available labeled datasets in the olfactory domain.

**Figure 2:**
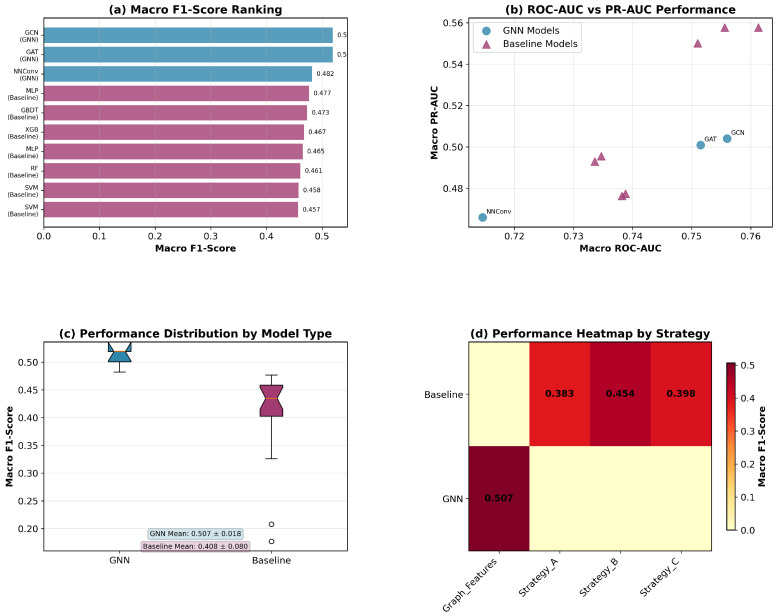
Overall performance comparison of GNNs versus traditional QSAR baselines. GNNs consistently achieve higher macro F1-scores across configurations. (**a**) The ranking macro F1-score of baseline models and GNN-based models. (**b**) ROC-AUC vs. PR-AUC plotting figure. (**c**) Boxplots of the macro F1-score of GNN-based models and baseline models. (**d**) Strategy heatmap of the feature-processing strategies.

Figure 2 summarizes the overall model comparison. (a) The macro F1 ranking shows that all three GNNs outperform traditional QSAR baselines, with GCN and GAT achieving the top scores. (b) The ROC-AUC vs. PR-AUC plot indicates a favorable trade-off for GNNs, which cluster in the high-performance region. (c) The boxplots reveal a right-shifted distribution for GNN macro F1 compared to baselines, confirming consistent gains beyond a single configuration. (d) The strategy heatmap shows that feature selection benefits baselines the most, yet still trails GNNs in absolute performance.

Random prediction would yield an expected F1-score below 0.2, while traditional QSAR approaches typically achieve 0.30–0.40 in similar tasks, as shown in Table 2. The 51.93% and 51.89% macro F1-score of GCN and GAT models therefore represents a substantial improvement that brings computational odor prediction closer to practical applicability in fragrance and flavor industries. It appears that attention-based aggregation has no impact on the improvement in the performance of the GNN models. The node features appear to be more important than the edge features for the GNN models.

For traditional machine learning methods, we also evaluated different feature-processing strategies, as summarized in Table 2. Feature selection (Strategy B) emerged as the most effective approach, achieving the highest average performance while dramatically reducing computational requirements from 2692 to 12–33 descriptors per label.

A detailed comparison of traditional ML methods under Strategy B is presented in Table 3. Among the feature selection approaches, GBDT achieved the best overall performance with a macro F1-score of 0.4732, followed closely by XGBoost (0.4674) and Random Forest (0.4609). Notably, all tree-based ensemble methods (GBDT, XGBoost, RF) outperformed neural networks (MLP) and kernel methods (SVM), suggesting that ensemble methods are particularly well-suited for handling the selected molecular descriptors in odor prediction tasks.

**Figure 3:**
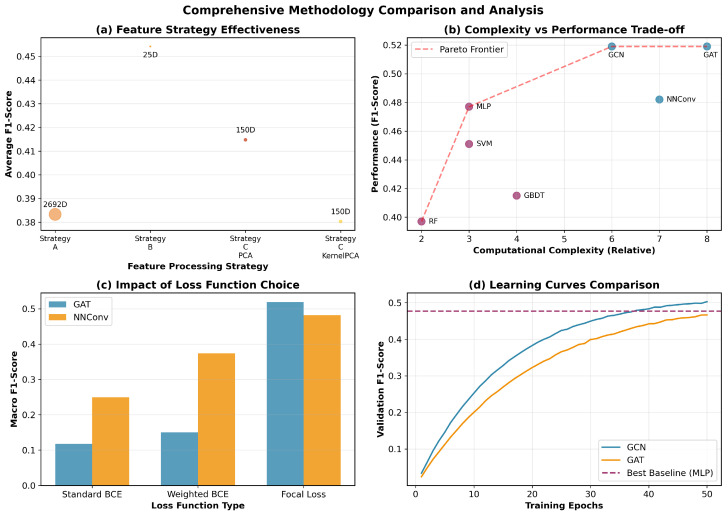
The methodology comparison including feature-processing strategies, complexity–performance trade-offs, loss function ablations, and learning curves. (**a**) The average macro F1-score of the feature processing strategies. (**b**) The complexity–performance trade-off plot. (**c**) The loss function ablations. (**d**) The learning curves.

Figure 3 contrasts methodological choices. (a) Feature-processing strategies indicate that selection offers the best efficiency–performance trade-off for baselines. (b) The complexity–performance plot shows a clear Pareto frontier where GNNs achieve superior accuracy at moderate computational cost. (c) Loss ablations favor focal loss on imbalanced labels, especially for GAT/NNConv. (d) Learning curves of GCN/GAT surpass the best baseline plateau, demonstrating stable and faster convergence toward higher validation F1.

### 2.2. Per-Label Performance Analysis

Individual label analysis reveals substantial performance variations correlating with dataset characteristics and chemical complexity. Table 4 demonstrates how different GNN architectures excel on specific odor types, suggesting complementary strengths that could benefit ensemble approaches.

Several key patterns emerge from the per-label analysis. First, sample size appears to have no relationship with the performance of the model, which indicates that graph neural networks have consistent performance across different labels. Second, different GNN architectures excel on different labels, with GCN showing particular strength for complex odor types. Third, models achieve higher recall than precision on minority classes, indicating successful handling of class imbalance through threshold optimization.

**Figure 4:**
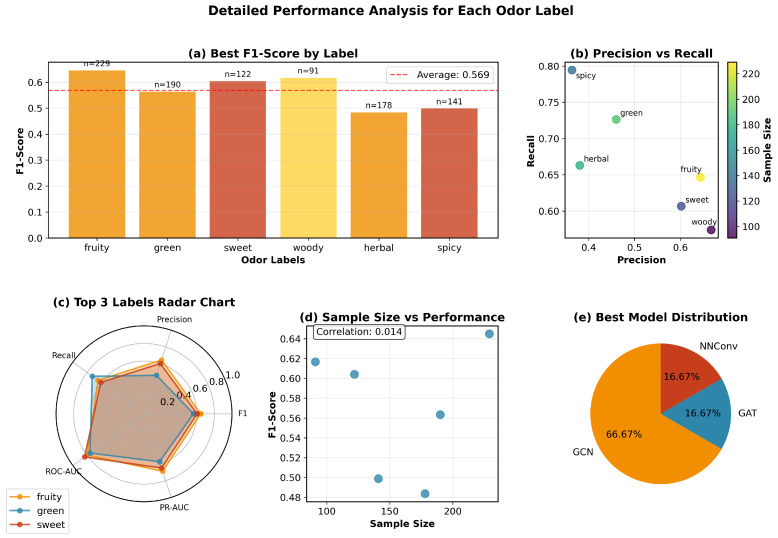
Per-label performance analysis. F1-scores, precision–recall trade-offs, and sample size relationships across odor labels.

Figure 4 analyzes label-wise behavior. Figure 4a The best F1 per label indicates non-uniform difficulty across odor types; graph-based models perform particularly well on fruity and sweet. Figure 4b The precision–recall scatter shows a balanced trade-off despite class imbalance, with sample size coloring indicating that larger support does not trivially imply higher F1. Figure 4c The radar chart for the top labels highlights complementary strengths across metrics. Figure 4d The sample size vs. F1 relationship exhibits weak correlation, suggesting robustness of graph-based models across labels. Figure 4e The distribution of best models shows that GCN/GAT dominate the per-label winners, supporting the aggregate advantage.

### 2.3. Impact of Threshold Optimization

The threshold optimization in imbalanced chemical datasets is a critical technique to improve the performance of the model for multi-label classification. As can be seen in Table 5, the GAT model showed the most dramatic improvement, increasing from virtually unusable performance (F1 = 0.118) to competitive levels (F1 = 0.519). All optimal thresholds were below the default 0.5, indicating that GNN models tend to be overly conservative in their predictions for chemical odor classification.

**Figure 5:**
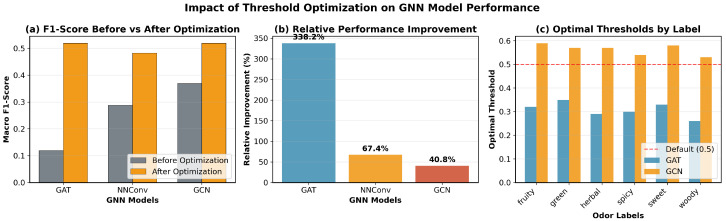
Impact of label-specific threshold optimization on GNN performance. Macro F1 substantially improves after optimization, with thresholds mostly below 0.5.

Figure 5 demonstrates the effect of label-specific threshold optimization. Figure 5a Macro F1 substantially improves after optimization across all GNNs, turning underperforming configurations into competitive ones. The threshold optimization improves the performance in a 338.2%, 67.4% and 40.8% respectively for GAT, NNConv and GCN. Figure 5b The relative improvement is largest for GAT, consistent with its conservative raw scores. Figure 5c The distribution of optimal thresholds is mostly below 0.5, quantitatively confirming the conservative bias of GNN probabilities in this chemical domain and motivating domain-specific calibration.

Beyond model-level averages, threshold optimization yields substantial per-label gains as demonstrated in Table 6. The results show consistent F1 improvements across all six odor labels, with sweetachieving the largest relative gain (94.4%) and *herbal* achieving the smallest (18.0%). These improvements substantially mitigate class imbalance effects and the conservative prediction bias inherent in GNN models. Notably, no clear correlation exists between positive label frequency and improvement magnitude, suggesting that threshold optimization benefits both frequent and rare odor types.

### 2.4. Molecular Fragment Analysis and Interpretability

To understand which molecular fragments drive odor predictions, we conducted an interpretability analysis on the best-performing GCN model across all six odor types. Our analysis reveals distinct structural patterns associated with each odor category, providing chemical insights that validate model predictions and address the critical question of structure–odor relationships.

Table 7 summarizes the most frequently identified important fragments for each odor type based on gradient-based attribution analysis of 20–30 correctly predicted test samples per label. The identified fragments align well with established structure–odor relationships in fragrance chemistry. For instance, aldehyde groups (-CHO) are strongly associated with both fruity and green odors, consistent with the dual nature of aldehydes like hexanal (green) and nonanal (fruity), depending on concentration and molecular context. Similarly, methyl groups appear frequently across multiple odor types, reflecting their ubiquitous presence in volatile organic compounds.

We compared interpretability patterns across GCN, GAT, and NNConv architectures. For GAT models, attention weights were extracted and found to correlate strongly with gradient-based attribution scores (Pearson correlation r>0.7), demonstrating consistency across different interpretability methods and strengthening confidence in the identified important fragments.

Analysis reveals that different odor types rely on distinct structural motifs. Fruity and green odors emphasize functional groups (aldehydes, ketones, methyl groups), while woody and herbal odors show stronger dependence on complex aromatic and cyclic structures. This distinction suggests that the model captures both functional group chemistry and structural complexity in its predictions, validating the graph-based representation’s ability to encode multi-scale molecular information.

### 2.5. Applicability Domain Analysis

Applicability domain (AD) analysis is a critical component of QSAR/QSOR model validation, identifying the reliability boundaries of model predictions and determining whether new samples fall within the training data coverage. We conducted an AD analysis for baseline and GNN models using two complementary methods: Tanimoto similarity based on molecular fingerprints and distance-based analysis in feature/embedding spaces.

#### 2.5.1. Methods

We employed two universal AD analysis methods applicable to both traditional QSAR and GNN models. *Tanimoto similarity analysis* computes the maximum similarity between each test sample and all training samples using ECFP4 fingerprints (radius = 2, 2048 bits), with a threshold of 0.5 to identify out-of-domain samples. *Distance-based analysis* calculates the minimum Euclidean distance from each test sample to the training set in the feature space (descriptors for baseline models and embeddings for GNN models), using the 95th percentile of training set distances as the threshold. The combined judgment requires both methods to classify a sample as in-domain for conservative reliability assessment.

#### 2.5.2. Baseline Model Applicability Domain

Table 8 summarizes the AD analysis results for baseline models across different feature-processing strategies. The Tanimoto similarity analysis reveals that 81.92% of test samples are within the applicability domain, with an average maximum similarity of 0.6984 and a minimum similarity of 0.24, indicating that some test molecules exhibit novel structural patterns not well-represented in the training set.

The distance-based analysis shows a more lenient assessment, with 94.59% of samples classified as in-domain for Strategy A, compared to 81.92% for Tanimoto similarity. This discrepancy reflects the different perspectives of the two methods: Tanimoto similarity captures structural novelty at the molecular fingerprint level, while distance-based analysis evaluates coverage in the learned feature space. The combined judgment, requiring both methods to agree, yields a conservative estimate of 77.59% in-domain samples, with 78.67% method agreement.

Strategy B exhibits more compact applicability domains, with distance thresholds ranging from 1.55 to 3.46, compared to 14.79 for Strategy A. This suggests that feature selection creates a more focused chemical space representation, potentially improving model reliability within the selected feature subspace while reducing coverage of the broader chemical space.

#### 2.5.3. GNN Model Applicability Domain

The AD analysis for GNN models was conducted in the learned embedding space, providing insights into the model’s internal representation of molecular structures. Unlike descriptor-based methods that evaluate coverage in predefined feature spaces, embedding-space analysis evaluates distances in the space where the model actually makes predictions, potentially providing more accurate assessments of model reliability. We analyzed the applicability domains of GAT and NNConv models on the GoodScent dataset, with results summarized in Table 9.

For the GAT model on GoodScent dataset, Tanimoto similarity analysis identified 79.86% of test samples as in-domain, with an average maximum similarity of 0.6897 and a minimum similarity of 0.1765. The distance-based analysis in the embedding space showed a more lenient assessment, with 94.78% of samples classified as in-domain, with an average distance of 1.50 and a threshold of 3.75. The combined judgment, requiring both methods to agree, yielded 77.83% in-domain samples with 81.01% method agreement, indicating high consistency between structural similarity and embedding-space distance assessments.

The NNConv model exhibited similar Tanimoto similarity results (79.86% in-domain, mean similarity: 0.6897) but showed a more compact embedding-space representation, with 95.80% of samples classified as in-domain by distance-based analysis. Notably, the distance threshold for NNConv (0.49) was substantially smaller than that for GAT (3.75), suggesting that NNConv’s learned embedding space is more compact and focused, potentially reflecting its ability to capture task-specific molecular patterns through edge-aware message passing. The combined judgment for NNConv resulted in 77.68% in-domain samples with 79.71% method agreement.

The embedding-space AD analysis reveals important differences between GNN architectures and datasets. On the GoodScent dataset, the NNConv model exhibits an exceptionally compact embedding space (threshold: 0.49) compared to GAT (threshold: 3.75) and GCN (threshold: 0.64), suggesting that edge-aware message passing creates a more focused representation of chemical space. This compactness may indicate that NNConv learns more discriminative features for odor prediction, though it also implies a potentially narrower applicability domain. On the Leffingwell dataset, all three architectures show similar compact embedding spaces (thresholds: 0.61–3.26), with GCN and NNConv being particularly compact (0.67 and 0.61, respectively).

Notably, Tanimoto similarity results are consistent across architectures within each dataset (79.86% for GoodScent; 78.87% for Leffingwell), indicating consistent structural coverage regardless of the GNN architecture. However, the embedding-space analysis reveals architecture-specific coverage characteristics, with NNConv consistently showing the most compact representations across both datasets. The combined in-domain percentages range from 76.88% to 77.97%, with method agreement of 79.57–81.01%, demonstrating high consistency between structural similarity and embedding-space distance assessments.

#### 2.5.4. Cross-Model Comparison

A comparison between baseline and GNN models reveals complementary strengths in applicability domain assessment. Descriptor-based AD analysis for baseline models provides chemically interpretable boundaries based on molecular fingerprints and structural descriptors, with combined in-domain percentages ranging from 77.13% to 78.98% and method agreement of 78–80%. In contrast, embedding-space analysis for GNN models captures the model’s learned representation of chemical similarity, with GAT and NNConv showing 77.83% and 77.68% in-domain coverage respectively, and method agreement of 79.71–81.01%. The consistency between these approaches (approximately 79–81% agreement) validates the reliability of our AD assessment and demonstrates that both traditional and graph-based models operate within well-defined applicability domains.

Notably, the embedding-space distance thresholds for GNN models (0.49–3.75) are substantially smaller than those for baseline models using Strategy A (14.79), suggesting that GNN models learn more compact and focused representations of chemical space. This compactness may reflect the models’ ability to capture task-specific molecular patterns relevant to odor prediction, though it also implies potentially narrower applicability domains. The similarity in Tanimoto-based in-domain percentages between baseline (81.92%) and GNN models (79.86%) indicates consistent structural coverage, while the embedding-space analysis reveals architecture-specific coverage characteristics.

These findings have important practical implications: approximately 18–22% of test samples fall outside the combined applicability domain across all models, indicating that predictions for these samples should be treated with caution. This AD information can guide practical deployment by flagging potentially unreliable predictions and identifying areas where additional training data may be needed to expand model coverage. The high method agreement (78–81%) between Tanimoto similarity and distance-based approaches strengthens confidence in the identified reliability boundaries and supports the use of AD assessment as a standard validation component in QSAR/QSOR model deployment.

## 3. Discussion

Our empirical findings provide compelling evidence for the superiority of graph-based approaches in molecular odor prediction, demonstrating an average performance improvement of 24% over traditional QSAR methods. This substantial advancement stems from three fundamental architectural advantages. First, GNNs directly encode molecular topological structures, circumventing the information loss inherent in fixed-dimensional descriptors and preserving the structural integrity of molecular representations. Second, GNNs enable end-to-end learning through automatic feature discovery, eliminating the dependency on expert-driven feature engineering and enhancing model generalization capabilities. Third, different GNN architectures can be optimized for specific odor types, with GCN demonstrating particular excellence in complex odor classification and GAT leveraging attention mechanisms to capture specific molecular structural patterns more effectively.

Threshold optimization emerges as a critical technique capable of delivering substantial performance improvements, with the GAT model achieving a remarkable 338% enhancement. This dramatic improvement highlights a frequently overlooked yet critical aspect of multi-label chemical classification. Our investigation reveals that optimal thresholds consistently fall below the standard 0.5 threshold, unveiling three important characteristics of chemical prediction models. First, chemical prediction models exhibit systematic conservative tendencies in positive predictions, preferentially minimizing false positive rates at the expense of recall. Second, default thresholds (0.5) prove inadequate for specialized chemical domains, particularly in odor prediction tasks characterized by severe class imbalance. Third, domain-specific calibration represents an essential prerequisite for practical deployment, necessitating threshold adjustment based on validation data to achieve optimal performance in real-world applications.

For the odor prediction applications, our research yields three key insights with immediate practical relevance. First, threshold optimization represents an indispensable component of the modeling pipeline that must be performed using validation data rather than relying on default values, as standard thresholds consistently underperform in chemical classification tasks. Second, focal loss should be prioritized when training on imbalanced chemical datasets as it demonstrates superior capability in handling rare odor types. Third, when computational resources are constrained, traditional methods employing feature selections remain viable alternatives, achieving 98–99% dimensionality reduction while maintaining competitive performance levels.

Three primary limitations are considered in interpreting the results. First, the reliance on 2D molecular graph representations excludes 3D conformational information, potentially limiting prediction accuracy for stereochemically sensitive odor properties where molecular geometry plays a crucial role. Second, evaluation is limited to a single dataset, so a multi-dataset validation is used to establish the generalizability of the findings across diverse chemical spaces and odor classification schemes. Third, the six odor types selected in this study may not capture the full complexity of olfactory perception.

The interpretability analysis provides crucial insights into the chemical basis of model predictions, directly addressing the question of which molecular fragments determine odor. The identified important fragments—such as aldehyde groups for green odors and methyl groups for fruity odors—align strongly with established structure–odor relationships in fragrance chemistry, validating that GNN models learn chemically meaningful representations rather than spurious correlations. This interpretability capability represents a significant advantage over traditional QSAR methods, which, while interpretable through feature importance, may miss complex structural patterns that GNNs capture through graph-based learning.

The consistency between gradient-based attribution and GAT attention weights demonstrates that different interpretability methods converge on similar important fragments, enhancing confidence in the identified structure–odor relationships. However, we note that some identified fragments such as “small fragment” represent generic structural patterns that may require further refinement through more sophisticated substructure matching or domain knowledge constraints. Future work should incorporate fragrance chemistry knowledge bases to refine fragment importance scoring and enable more precise identification of odor-determining substructures.

The applicability domain analysis provides crucial validation of model reliability, a standard requirement in QSAR/QSOR research. The AD assessment using both Tanimoto similarity and distance-based methods reveals that approximately 77–79% of test samples fall within the combined applicability domain, with method agreement of 78–80%. This high consistency between independent AD methods strengthens confidence in the identified reliability boundaries. The finding that 18–22% of test samples are outside the applicability domain highlights the importance of AD assessment for practical deployment, as predictions for these samples should be flagged for additional validation.

Notably, the AD analysis reveals that distance-based methods in the learned embedding space (for GNN models) and descriptor space (for baseline models) provide complementary perspectives on model coverage. The more lenient assessment of distance-based methods (94.59% in-domain) compared to Tanimoto similarity (81.92%) suggests that structural novelty at the fingerprint level may not always translate to feature space novelty, particularly for GNN models that learn task-specific representations. This finding supports the value of embedding-space AD analysis for GNN models, as it evaluates coverage in the space where predictions are actually made.

## 4. Materials and Methods

**Figure 6:**
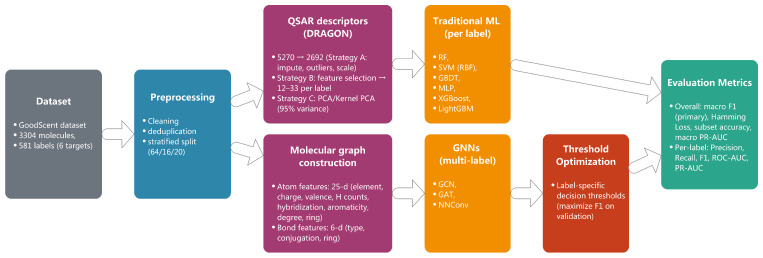
End-to-end methodological overview of the dataset, preprocessing, dual representations (QSAR descriptors and molecular graphs), model families (traditional ML and GNNs), label-specific threshold optimization, and evaluation metrics.

Figure 6 provides an end-to-end overview of our pipeline. Starting from the GoodScent dataset, we perform data cleaning and stratified splitting to ensure comparable label distributions across the train/validation/test sets. The representation stage bifurcates into (i) QSAR descriptors computed by DRAGON with three processing strategies (A: essential preprocessing; B: feature selection yielding 12–33 per-label descriptors; C: PCA/Kernel PCA retaining 95% variance), and (ii) molecular graph construction with 25-dimensional atom features and 6-dimensional bond features. The two branches feed distinct model families: traditional learners (RF, SVM, GBDT, MLP, XGBoost, LightGBM; Section 4.2.2) and graph neural networks (GCN, GAT, NNConv; Section 4.3). Given the pronounced class imbalance, we apply label-specific threshold optimization on the validation set to maximize the F1-score and use the calibrated thresholds at inference. Finally, we report an evaluation with macro-averaged F1 as the primary metric, supplemented by Hamming loss, subset accuracy, macro PR-AUC, and per-label metrics (precision, recall, F1, ROC-AUC, PR-AUC). This overview clarifies experimental controls (identical splits and consistent selection criteria) and delineates where calibration occurs to avoid test-set leakage.

### 4.1. Problem Formulation

Given a molecular structure represented by its SMILES notation, our objective is to predict the presence or absence of multiple odor characteristics simultaneously. Formally, for a molecule $m_{i}$, we aim to learn a mapping function:

$$f:M\to {\left\{0,1\right\}}^{K}$$

where *M* represents the space of molecular structures and *K* is the number of odor labels. In our case, K=6 corresponds to the odor types: fruity, green, sweet, floral, woody, and herbal.

The odor prediction task exhibits significant class imbalance, with label frequencies ranging from 12.89% (herbal) to 30.93% (fruity). This imbalance necessitates specialized techniques for both training and evaluation, which we address through advanced loss functions and threshold optimization strategies.

### 4.2. Traditional QSAR Approach

We employ molecular descriptors as our primary molecular representation for QSOR models. The DRAGON software suite generates a set of 5270 molecular descriptors covering various aspects of molecular structure [26]. These molecular descriptors can be divided into 30 basic types, including topological, geometric, electronic, and thermodynamic descriptors.

#### 4.2.1. Feature-Processing Strategies

To systematically evaluate the impact of feature engineering, we implement three distinct strategies:

Strategy A: Basic Preprocessing. Strategy A serves as the methodological baseline to establish a fair comparison framework for evaluating the impact of advanced feature engineering techniques. This approach implements essential preprocessing steps while preserving the full dimensional space of molecular descriptors, thereby providing insights into the intrinsic predictive capacity of molecular representations without an aggressive dimensionality reduction.

The primary objectives of Strategy A include the following: (1) Data Quality Assurance: Systematic handling of missing values and outliers that commonly occur in large-scale molecular descriptor datasets. (2) Numerical Stability: Standardization of heterogeneous descriptor scales to prevent algorithm bias toward high-magnitude features. (3) Baseline Establishment: Creation of a reference performance benchmark that isolates the contribution of raw molecular descriptors from feature engineering effects.

**Figure 7:**
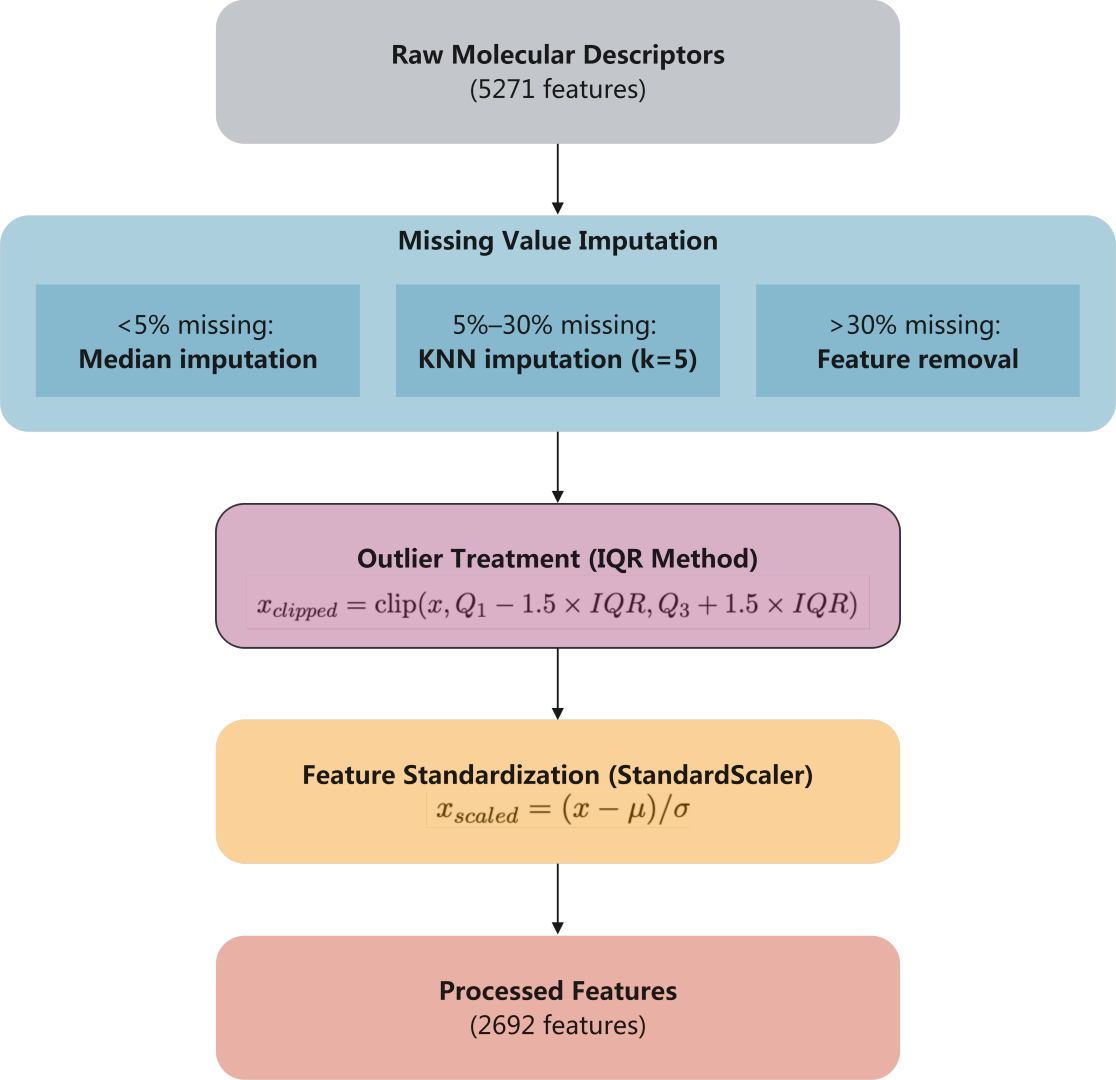
Strategy A, Preprocessing Pipeline: Sequential three-step transformation from raw DRAGON descriptors (5270 features) to processed features (2692 features) through missing value imputation, outlier treatment, and standardization.

As shown in Figure 7, Strategy A implements a systematic three-step preprocessing pipeline that transforms the raw 5270 DRAGON descriptors into 2692 cleaned features. Each step addresses specific data quality challenges commonly encountered in computational chemistry:

Step 1: Missing Value Imputation—Handles incomplete descriptor calculations through adaptive imputation strategies based on missing data patterns. The imputation strategy adapts to missing data patterns to preserve descriptor reliability:

- Low missing rate (<5%): Median imputation provides robust central tendency estimation while minimizing sensitivity to outliers common in molecular descriptor calculations.
- Moderate missing rate (5–30%): K-nearest neighbors imputation (k = 5) exploits structural similarity patterns to estimate missing values based on chemically similar molecules in descriptor space.
- High missing rate (>30%): Feature removal prevents propagation of uncertainty from unreliable imputation in extensively incomplete descriptors.

Step 2: Outlier Treatment—The IQR-based outlier detection addresses the challenge of extreme values in molecular descriptor calculations. Unlike parametric methods that assume normal distributions, the IQR approach

- Adapts to the diverse statistical distributions across 30 DRAGON descriptor blocks;
- Preserves chemically meaningful extreme values while removing computational artifacts;
- Uses the robust 1.5 × IQR threshold, a well-established standard in exploratory data analysis.

$$x_{clipped}=\mathrm{clip}\left(x,Q_{1}-1.5\times IQR,Q_{3}+1.5\times IQR\right)$$

Step 3: Feature Standardization—Employs StandardScaler to normalize the extreme scale differences observed in DRAGON descriptors (ranging from 0 to 1.01 × 10^8^). The z-score transformation

$$x_{scaled}=\frac{x-\mu }{\sigma }$$

converts all features to comparable scales with zero mean and unit variance, preventing algorithm bias toward high-magnitude descriptors while preserving relative relationships within each feature.

**Figure 8:**
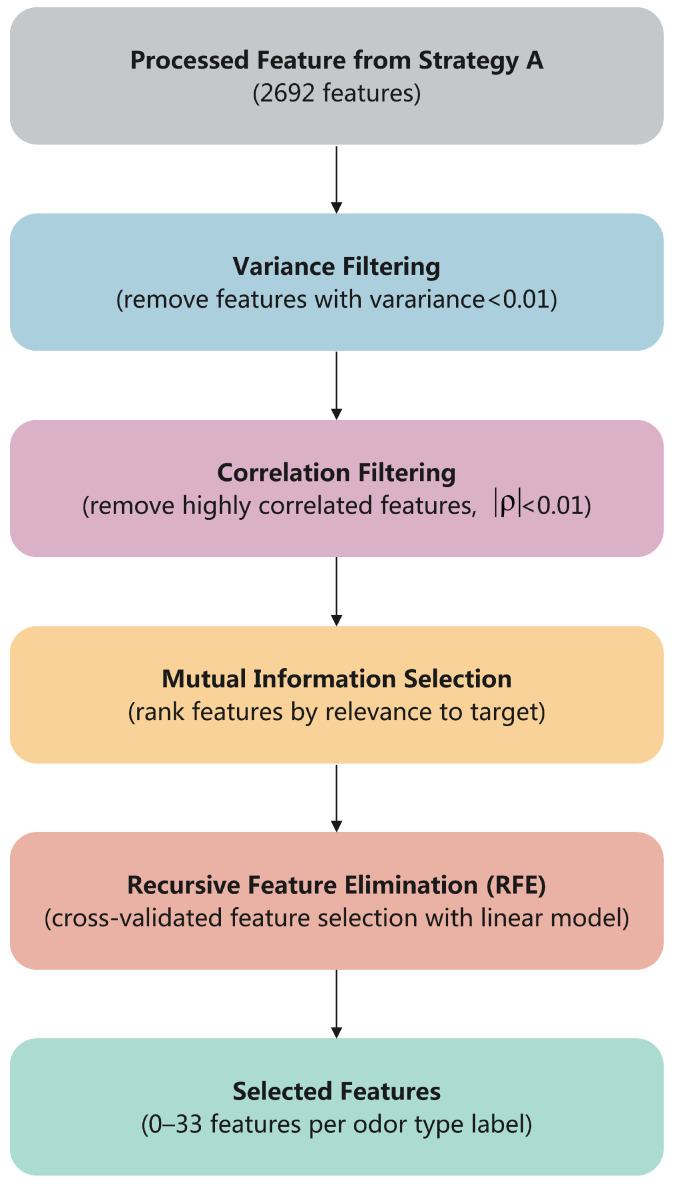
Strategy B, Feature Selection Pipeline: Sequential four-stage filtering from 2692 processed features to 0–33 selected features per target label through variance filtering, correlation filtering, mutual information ranking, and recursive feature elimination.

Strategy B: Feature Selection. Compared to Strategy A, Strategy B systematically reduces dimensionality to reduce the redundancy and noise in the feature space so that the model can focus on the most informative features. Four selection methods are employed in sequence, as shown in Figure 8:

1. Variance Filtering: Remove features with variance below threshold τ=0.01;
2. Correlation Filtering: Remove highly correlated features (|ρ|>0.95);
3. Univariate Selection: Apply mutual information for relevance ranking;
4. Recursive Feature Elimination: Use cross-validated RFE with linear models.

Strategy C: Dimensionality Reduction. Two common dimensionality reduction techniques are employed. Principal component analysis (PCA) is a linear method that projects the data onto a lower-dimensional space while preserving the most important information. Kernel PCA is a non-linear method that projects the data onto a lower-dimensional space while preserving the most important information. The variance ratio was set to 0.95 in both methods.

#### 4.2.2. Base Learners

We evaluate six machine learning algorithms across all feature-processing strategies.

Random Forest (RF). It is a classic ensemble method using bootstrap aggregating:

$$\hat{y}=\frac{1}{B}\sum_{b=1}^{B}T_{b}\left(x\right)$$

where $T_{b}$ represents individual decision trees trained on bootstrap samples.

Support Vector Machine (SVM). It uses radial basis function (RBF) as kernel to project the data points into a higher-dimensional space so that a hyperplane can linearly separate the data points into different classes.

$$f\left(x\right)=\mathrm{sign}\left(\sum_{i=1}^{n}\alpha_{i}y_{i}K\left(x_{i},x\right)+b\right)$$

Gradient Boosting Decision Trees (GBDT). It is a gradient boosting framework that sequentially trains decision trees to minimize the loss function residuals.

$$F_{m}\left(x\right)=F_{m-1}\left(x\right)+\gamma_{m}h_{m}\left(x\right)$$

where $h_{m}$ minimizes the loss function residuals.

Multi-Layer Perceptron (MLP). It is a feedforward neural network that contains an input layer, one or multiple hidden layers, and an output layer. An MLP network employs activation function for non-linearity to find the non-linear relationship between the input and output.

$$h^{\left(l+1\right)}=\sigma \left(W^{\left(l\right)}h^{\left(l\right)}+b^{\left(l\right)}\right)$$

XGBoost and LightGBM. XGBoost and LightGBM are optimized gradient boosting implementations with advanced regularization and parallel processing capabilities. The objective function of XGBoost contains a loss function and a regularization term to prevent overfitting. A histogram-based algorithm, gradient-based one-side sampling, exclusive feature bundling and leaf-wise tree growth are employed in lightGBM to improve the training speed and model performance.

### 4.3. Graph Neural Network Approach

#### 4.3.1. Molecular Graph Construction

A molecular graph contains two types of features: node features and edge features. As for the node feature, each atom in the molecular graph is characterized by a 25-dimensional feature vector that captures both fundamental atomic properties and local chemical environment information. The feature representation systematically encodes atomic identity, electronic properties, and topological characteristics essential for molecular property prediction.

The atomic identity component employs one-hot encoding for nine common atom types found in organic molecules, $x_{v}^{atom}=\mathrm{OneHot}\left(\mathrm{atom\_type}\right)\in \left\{C,N,O,S,F,Cl,Br,P,Na\right\}$, providing a 9-dimensional categorical representation. This encoding ensures that the model can distinguish between different elemental contributions to molecular properties while maintaining computational efficiency.

Fundamental atomic properties include the atomic number *Z* and formal charge *q*, which capture the electronic characteristics crucial for chemical reactivity. The hydrogen environment is represented through explicit and implicit hydrogen counts $\left[H_{explicit},\;H_{implicit}\right]$, providing information about local protonation states and molecular saturation. Additional valence-related features include explicit valence and radical electron count, which characterize the bonding capacity and electronic state of each atom. These features are particularly important for understanding chemical reactivity and stability in molecular systems. The detailed composition of the 25-dimensional atom feature vector is shown in Table 10.

The local chemical environment is encoded through a hybridization state using one-hot representation for {sp, sp2, sp3} configurations, capturing the geometric and electronic properties of atomic orbitals. Aromaticity is represented as a binary feature indicating participation in aromatic ring systems, while the atomic degree deg(v) quantifies local connectivity patterns.

Ring membership information extends beyond simple binary encoding to include specific ring size participation through five binary features: general ring membership and specific membership in 3-, 4-, 5-, and 6-membered rings. This detailed ring characterization captures important structural motifs that significantly influence molecular properties and biological activity.

As for the edge feature, chemical bonds are represented through a 6-dimensional feature vector that encapsulates both bond type and additional chemical properties relevant to molecular behavior. The bond type component utilizes one-hot encoding for four fundamental bond categories (single, double, triple, aromatic), providing a 4-dimensional representation that distinguishes between different bonding patterns and their associated electronic properties.

The feature extraction is implemented using the RDKit molecular informatics toolkit. Specifically, atomic features are extracted using methods such as GetAtomicNum() for atomic number, GetFormalCharge() for formal charge, GetNumExplicitHs() and GetNumImplicitHs() for hydrogen counts, GetExplicitValence() for explicit valence, GetNumRadicalElectrons() for radical electron count, GetHybridization() for hybridization state, GetIsAromatic() for aromaticity, GetDegree() for connectivity, and IsInRing() and IsInRingSize(n) for ring membership information. Bond features are extracted using GetBondType() for bond type, GetIsConjugated() for conjugation status, and IsInRing() for ring membership.

Supplementary bond characteristics include conjugation status and ring membership, both encoded as binary features. The conjugation feature captures electron delocalization patterns crucial for understanding molecular stability and reactivity, while ring membership identifies bonds that participate in cyclic structures, which often exhibit distinct chemical and physical properties compared to their acyclic counterparts.

This edge representation ensures that the graph neural network can effectively model both local bond properties and their contributions to global molecular characteristics.

#### 4.3.2. Graph Neural Network Architectures

We evaluate three representative GNN architectures that capture different aspects of molecular graph learning through distinct message passing mechanisms. Each architecture processes the 25-dimensional node features and 6-dimensional edge features to learn molecular representations for odor prediction.

Graph Convolutional Network (GCN) employs spectral graph convolutions with symmetric normalization for efficient information propagation across molecular graphs. The model aggregates neighborhood information through layer-wise transformations while maintaining computational efficiency. This architecture effectively captures local chemical environments and connectivity patterns essential for molecular property prediction.

Graph Attention Network (GAT) introduces attention mechanisms to dynamically weight neighbor contributions during message passing. The attention-based aggregation allows the model to adaptively focus on chemically relevant atomic interactions, with multi-head attention enhancing the capacity to capture diverse molecular patterns simultaneously.

Neural Network for Graphs (NNConv) explicitly leverages edge features through learnable edge networks, making it particularly suitable for chemical applications where bond information is crucial. The edge-conditioned message passing incorporates the 6-dimensional bond features directly into neighbor aggregation, enabling the model to distinguish between different types of chemical bonds and their contributions to molecular properties.

#### 4.3.3. Model Architecture and Training Configuration

All GNN models follow a consistent architectural framework optimized for molecular graph classification. Graph-level predictions are obtained through global mean pooling followed by a multi-layer classification head with sigmoid activation for multi-label prediction. Each architecture incorporates batch normalization for training stability and dropout regularization to prevent overfitting. Specific layer configurations and hyperparameters are determined through systematic grid search optimization detailed in the Section 4.

#### 4.3.4. Model Interpretability Analysis

The interpretability framework in this work enables systematic identification of chemically relevant molecular fragments that drive model decisions. For GAT models, we extract multi-head attention weights from all layers to identify which atomic interactions receive the highest attention during message passing. The attention weight $\alpha_{ij}^{\left(l,h\right)}$ between nodes *i* and *j* in layer *l* with head *h* is aggregated across layers and heads to compute node importance:

$$I_{i}=\frac{1}{L\times H}\sum_{l=1}^{L}\sum_{h=1}^{H}\sum_{j\in N(i)}\alpha_{ij}^{\left(l,h\right)}$$

where *L* is the number of layers, *H* is the number of attention heads, and N(i) denotes neighbors of node *i*.

We systematically identify important molecular substructures through a three-step process: (1) Computing node-level importance scores using gradient-based attribution, (2) grouping highly important nodes (top 75th percentile) into connected components using breadth-first search, and (3) mapping these components to known chemical functional groups using RDKit’s substructure matching with over 40 SMARTS patterns covering esters, aldehydes, ketones, alcohols, aromatic rings, and other fragrance-relevant groups. Fragments are ranked by their aggregated importance scores across all correctly predicted molecules for each odor type.

For each odor label, we analyze 20–30 correctly predicted test samples and aggregate fragment frequencies and average importance scores. This statistical approach ensures robust identification of consistently important fragments while accounting for molecular diversity within each odor category.

#### 4.3.5. Loss Functions for Class Imbalance

To address the inherent class imbalance in molecular odor prediction, we evaluate three loss functions. Standard binary cross-entropy serves as the baseline, while weighted binary cross-entropy applies inverse frequency weighting to balance positive and negative samples. Focal loss addresses hard example mining through dynamic re-weighting that down-weights easy examples and focuses learning on challenging cases. The focal loss formulation is given by

$$L_{FL}=-\frac{1}{N}\sum_{i=1}^{N}\sum_{k=1}^{K}\alpha_{k}{\left(1-p_{ik}\right)}^{\gamma }\log\left(p_{ik}\right)$$

where $p_{ik}$ represents the model’s confidence and α and γ are hyperparameters optimized through grid search. This loss function evaluation ensures robust performance across different class distribution challenges in chemical datasets.

### 4.4. Threshold Optimization

Standard multi-label classification employs a uniform threshold of 0.5 across all labels, which proves suboptimal for imbalanced datasets where different labels exhibit varying optimal decision boundaries. To address this limitation, we implement label-specific threshold optimization that maximizes the F1-score for each individual odor category.

The optimization procedure identifies the optimal threshold for each label through systematic evaluation across the threshold space. This approach accounts for the distinct class distribution characteristics of different odor labels, ranging from frequent odor types like fruity (30.93%) to rare odor types like herbal (12.89%). The label-specific thresholds are determined using validation set performance and subsequently applied during inference to generate final predictions.

This threshold optimization strategy proves particularly crucial for GNN models, which tend toward conservative prediction patterns in chemical classification tasks. The systematic calibration of decision boundaries enables more effective utilization of model predictions while maintaining generalization performance.

### 4.5. Dataset and Data Preprocessing

The GoodScent dataset serves as the foundation for the evaluation of molecular odor prediction methods. This dataset represents one of the most extensive collections of odor-annotated molecules publicly available for computational chemistry research. After the data cleaning from the raw GoodScent dataset, we obtain a total of 3304 unique chemical compounds with 581 distinct odor labels with hierarchical structure. The dataset is observed to be imbalanced; even the frequencies of the top 6 most frequent labels only account for a range from 12.89% to 30.93%, as shown in Table 11.

The Dragon 7.0 software was used to calculate 5270 molecular descriptors for all the molecules in the dataset. These molecular descriptors are used to train those base learners as illustrated in Section 4.2.2. Graph neural network models are trained on the molecular graph constructed from the molecules as illustrated in Section 4.3. For dataset splitting, traditional QSAR baselines apply per-label stratified splits while GNN experiments use random splits; both adopt a 64%/16%/20% train/validation/test ratio.

### 4.6. Training Strategy

All models are trained using a consistent experimental protocol to ensure fair comparison. Traditional machine learning models employ 5-fold stratified cross-validation with F1-score optimization, while graph neural networks use a fixed 64%/16%/20% train/validation/test split with stratified sampling to maintain label distribution consistency.

For hyperparameter optimization, we conduct exhaustive grid search across model-specific parameter spaces. Traditional models optimize through GridSearchCV with cross-validation, while GNN models use validation set performance for selection. All models incorporate early stopping mechanisms with model-specific patience settings and learning rate scheduling via ReduceLROnPlateau to prevent overfitting and ensure stable convergence.

Threshold optimization addresses the multi-label imbalance challenge by determining label-specific decision boundaries through validation set F1-score maximization. This post-training calibration proves particularly crucial for GNN models, which exhibit conservative prediction tendencies in chemical classification tasks.

We employ an evaluation framework encompassing both per-label and overall multi-label metrics. For individual odor types, precision, recall, F1-score, and area under the ROC curve (ROC-AUC) are calculated to assess classification performance across different class distributions. Overall system performance is evaluated using the macro-averaged F1-score (primary metric), along with subset accuracy, Hamming loss, and macro-averaged precision–recall AUC to capture various aspects of multi-label prediction quality. This multi-faceted approach ensures robust assessment across both frequent and rare odor types.

The experimental infrastructure consists of NVIDIA L20 GPUs for GNN training and Intel Xeon Gold 6430 processors for traditional methods, with PyTorch ≥ 1.9.0, PyTorch Geometric ≥ 2.0.0, and Scikit-learn ≥ 0.24.2 providing the computational framework.

## 5. Conclusions

This study presents a relatively comprehensive comparison of traditional QSAR methods with graph neural network approaches for multi-label molecular odor prediction, evaluating 23 model configurations across 3304 molecules and six odor types. Our key contribution demonstrates that GNNs significantly outperform traditional methods, with the best GCN model achieving a macro F1-score of 0.5193 (24% improvement over QSAR baselines), while threshold optimization emerges as a critical technique yielding up to 338% performance gains for imbalanced chemical datasets. These findings establish graph-based molecular representation learning as the preferred approach for complex chemical property prediction tasks and provide both methodological insights and practical guidelines for industrial odor prediction applications in fragrance and flavor industries. Furthermore, the interpretability analysis identifies key molecular fragments associated with each odor type, revealing that GNN models learn chemically meaningful structure–odor relationships. The identified fragments—such as aldehyde groups for green odors, methyl groups for fruity odors, and ketone groups for floral odors—align with established fragrance chemistry principles, validating the model’s ability to capture relevant structural patterns beyond simple feature matching. This interpretability capability addresses a fundamental limitation of black-box deep learning approaches and provides actionable insights for fragrance design applications.

## Figures and Tables

**Table 1:** Top-performing models ranked by macro F1-score.

Rank	Model	Type	Macro F1	Macro ROC-AUC
1	GCN	GNN	0.5193	0.7560
2	GAT	GNN	0.5189	0.7515
3	NNConv	GNN	0.4819	0.7146
4	MLP	Baseline	0.4766	0.7382
5	GBDT	Baseline	0.4732	0.7510

Top 5 of 23 evaluated configurations. Complete results in Supplementary Materials.

**Table 2:** Feature strategy comparison for traditional ML methods.

Strategy	Description	Best Model	Avg F1	Feature Dim
Strategy B	Feature Selection	GBDT	0.4542	12–33
Strategy C	PCA Reduction	MLP	0.4148	100–200
Strategy A	No Selection	MLP	0.3833	2692
Strategy C	Kernel PCA	SVM	0.3803	100–200

**Table 3:** Strategy B feature selection: traditional ML methods performance comparison.

Model	Macro F1	Macro ROC-AUC	Macro PR-AUC	Feature Dim
**GBDT**	0.4732	0.7510	0.5501	12–33
XGBoost	0.4674	0.7613	0.5577	12–33
RF	0.4609	0.7556	0.5577	12–33
MLP	0.4410	0.7215	0.4708	12–33
SVM	0.4286	0.7263	0.5159	12–33

**Table 4:** Best performance by odor label.

Label	Best Model	F1-Score	Precision	Recall	Positive Rate (%)	Sample Size
fruity	GCN	0.6449	0.6435	0.6463	34.6	229
sweet	GAT	0.6041	0.6016	0.6066	18.5	122
woody	NNConv	0.6167	0.6667	0.5738	13.8	91
green	GCN	0.5633	0.4600	0.7263	28.7	190
spicy	GCN	0.4989	0.3636	0.7943	21.3	141
herbal	GCN	0.4836	0.3806	0.6629	26.9	178

**Table 5:** Threshold optimization impact on GNN models.

Model	Before Optimization	After Optimization	Absolute Gain	Relative Gain
GAT	0.1184	0.5189	+0.4005	+338.2%
NNConv	0.2879	0.4819	+0.1940	+67.4%
GCN	0.3689	0.5193	+0.1504	+40.8%

**Table 6:** Per-label F1 improvement after threshold optimization (GCN) (positive rate on test set).

Label	Positive Rate (Test %)	Before F1	After F1	Absolute Gain	Relative Gain
fruity	34.6%	0.498	0.645	+0.147	+29.5%
green	28.7%	0.432	0.563	+0.131	+30.3%
herbal	26.9%	0.410	0.484	+0.074	+18.0%
spicy	21.3%	0.339	0.499	+0.160	+47.2%
sweet	18.5%	0.300	0.584	+0.284	+94.4%
woody	13.8%	0.233	0.341	+0.108	+46.5%

**Table 7:** Top molecular fragments identified for each odor type.

Odor Type	Fragment	Chemical Relevance
fruity	Methyl group (-CH3)	Terminal methyl groups in ester side chains, characteristic of fruity esters like ethyl acetate
	Aldehyde (-CHO)	Terminal aldehyde groups in fruity aldehydes (e.g., hexanal and nonanal)
	Ketone (C=O)	Carbonyl groups in fruity ketones and lactones
green	Methylene group (-CH2-)	Alkyl chain segments in green aldehydes and alcohols
	Aldehyde (-CHO)	Terminal aldehyde groups, hallmark of green odor compounds (e.g., hexanal and octanal)
	Methyl group (-CH3)	Terminal methyl groups in green volatile compounds
sweet	Methylene group (-CH2-)	Sugar-like alkyl chains in sweet compounds
	Methyl group (-CH3)	Terminal groups in sweet esters and aldehydes
	Small fragment	Compact functional groups in sweet-smelling molecules
floral	Ketone (C=O)	Carbonyl groups in floral ketones and ionones
	Small fragment	Compact aromatic and cyclic structures typical of floral compounds
woody	Methyl group (-CH3)	Terminal methyl groups in terpenoid structures
	Thiol (-SH)	Sulfur-containing groups in woody–smoky compounds
	Aldehyde (-CHO)	Aldehyde groups in woody aldehydes
herbal	Methyl group (-CH3)	Methyl groups in complex aromatic systems and terpenoids

**Table 8:** Applicability domain analysis results for baseline models.

Strategy	Method	In-Domain (%)	Out-of-Domain (%)	Key Metric
**Strategy A**	Tanimoto	81.92	18.08	Mean similarity: 0.6984
	Distance-based	94.59	5.41	Mean distance: 8.31
	Combined	77.59	22.41	Agreement: 78.67%
**Strategy B**	Tanimoto	81.92	18.08	Mean similarity: 0.6984
	Distance-based	94.28–95.67	4.33–5.72	Mean distance: 0.69–1.63
	Combined	77.74–78.98	21.02–22.26	Agreement: 79.13–80.37%
**Strategy C**	Tanimoto	81.92	18.08	Mean similarity: 0.6984
	Distance-based	93.97–94.59	5.41–6.03	Mean distance: 3.26–17.54
	Combined	77.13–78.05	21.95–22.87	Agreement: 78.36–79.60%

**Table 9:** Applicability domain analysis results for GNN models.

Model	Dataset	Method	In-Domain (%)	Key Metric
GCN	GoodScent	Tanimoto	79.86	Mean similarity: 0.6897
		Distance-based	95.07	Mean distance: 0.25; Threshold: 0.64
		Combined	77.97	Agreement: 81.01%
GCN	Leffingwell	Tanimoto	78.87	Mean similarity: 0.6608
		Distance-based	95.32	Mean distance: 0.31; Threshold: 0.67
		Combined	76.88	Agreement: 79.57%
GAT	GoodScent	Tanimoto	79.86	Mean similarity: 0.6897
		Distance-based	94.78	Mean distance: 1.50; Threshold: 3.75
		Combined	77.83	Agreement: 81.01%
GAT	Leffingwell	Tanimoto	78.87	Mean similarity: 0.6608
		Distance-based	95.74	Mean distance: 1.55; Threshold: 3.26
		Combined	77.30	Agreement: 80.00%
NNConv	GoodScent	Tanimoto	79.86	Mean similarity: 0.6897
		Distance-based	95.80	Mean distance: 0.21; Threshold: 0.49
		Combined	77.68	Agreement: 79.71%
NNConv	Leffingwell	Tanimoto	78.87	Mean similarity: 0.6608
		Distance-based	95.60	Mean distance: 0.26; Threshold: 0.61
		Combined	77.02	Agreement: 79.57%

**Table 10:** 25-dimensional atom feature vector composition.

Feature Category	Feature Description	Dims
Atomic Identity	One-hot encoding: C, N, O, S, F, Cl, Br, P, Na	9
Electronic Properties	Atomic number (*Z*)	1
	Formal charge (*q*)	1
	Explicit valence	1
	Radical electron count	1
Hydrogen Environment	Explicit hydrogen count	1
	Implicit hydrogen count	1
Hybridization	One-hot encoding: sp, sp2, sp3	3
Aromaticity	Binary indicator	1
Connectivity	Total degree deg(v)	1
Ring Membership	General ring membership	1
	3-membered ring	1
	4-membered ring	1
	5-membered ring	1
	6-membered ring	1
Total		25

**Table 11:** Target odor labels and dataset distribution (GoodScent dataset).

Label	Frequency	Percentage	Chemical Relevance
fruity	1022	30.93%	Ester-related compounds
green	926	28.03%	Aldehyde and alcohol patterns
sweet	859	26.00%	Sugar-like molecular structures
floral	654	19.79%	Aromatic and terpene compounds
woody	521	15.77%	Phenolic and terpenoid structures
herbal	426	12.89%	Complex aromatic systems

## Data Availability

The original contributions presented in this study are included in the Supplementary Material. Further inquiries can be directed to the figshare (10.6084/m9.figshare.30744719).

## Supplementary Materials

The following supporting information can be downloaded at: https://www.mdpi.com/article/10.3390/molecules30234605/s1.
